# Reverse total shoulder arthroplasty in massive rotator cuff tears: does the Hamada classification predict clinical outcomes?

**DOI:** 10.1007/s00402-021-03755-w

**Published:** 2021-01-28

**Authors:** Max J. Kääb, Georges Kohut, Ulrich Irlenbusch, Thierry Joudet, Falk Reuther

**Affiliations:** 1 Sporthopaedicum Straubing, Bahnhofplatz 27, 94315 Straubing, Germany; 2 Orthopedics and Traumatology, Clinique Générale Ste-Anne, rue Hans-Geiler 6, 1700 Fribourg, Switzerland; 3 Sportklinik Erfurt, Am Urbicher Kreuz 7, 99099 Erfurt, Germany; 4 Clinique Chirurgicale du Libournais, Orthopedic Surgery Center, 119 rue de la Marne, 33500 Libourne, France; 5 DRK Kliniken Berlin Köpenick, Clinic for Trauma Surgery and Orthopedics, Salvador-Allende-Strasse 2-8, 12559 Berlin, Germany

**Keywords:** Hamada classification, Cuff tear arthropathy, Massive rotator cuff tears, Reverse total shoulder arthroplasty, Predictors, Midterm clinical outcome

## Abstract

**Introduction:**

Reverse total shoulder arthroplasty (RTSA) is a widely recognized treatment to reduce pain and improve shoulder function for patients in various disease stages of cuff tear arthropathy (CTA). However, it remains unclear whether outcomes after RTSA depend on the preoperative stage of CTA. Therefore, this study evaluated whether the Hamada classification influences midterm clinical outcomes after RTSA.

**Materials and methods:**

In this multicenter observational study, patients underwent inverted bearing RTSA for massive rotator cuff tears or CTA. Shoulders were grouped into those with (Hamada grades 4a, 4b, and 5) and those without (Hamada grades 1, 2, and 3) glenohumeral arthritis. Clinical outcomes, including range of motion, Constant score, American Shoulder and Elbow Surgeons score, and visual analog scale for pain and satisfaction, were determined preoperatively and at 24 and > 30 months. All complications were recorded, and survival free from any implant component revision was calculated.

**Results:**

Overall, 202 patients (211 shoulders) were treated with RTSA at a mean age of 75.8 ± 6.6 years (range 41.9–91.6 years). Of these, 144 patients (151 shoulders) were available for a mean follow-up of 79.9 ± 24.7 months (range 30.2–132.3 months). No significant between-group differences were found for clinical outcomes at 24 and > 30 months (*P* > 0.05). Furthermore, the Hamada classification did not correlate with clinical outcomes at 24 (*P* = 0.98) and > 30 months (*P* = 0.29). Revision-free implant component survival was similar between groups (*P* = 0.17). Postoperative complications were found in 11 shoulders, of which 10 required revision.

**Conclusions:**

Inverted bearing RTSA was found to be an effective treatment with similarly good midterm clinical outcomes, similar revision rates, and high implant survival rates in every stage of massive rotator cuff tears. Overall, the preoperative Hamada classification did not influence clinical outcomes or complications after RTSA.

## Introduction

Chronic rotator cuff tears can be classified according to several systems [1–3]. The Hamada classification system describes massive rotator cuff tear progression through a series of pathomechanical stages with accompanying radiographic changes [4, 5]. In the most severe stage, massive rotator cuff tears may lead to cuff tear arthropathy (CTA), a common shoulder pathology characterized by rotator cuff insufficiency, cranial migration of the humeral head, and arthritic changes of the subacromial space and the glenohumeral joint [4]. The Hamada classification may help orthopedic surgeons select more appropriate treatment for patients.

Reverse total shoulder arthroplasty (RTSA) is a widely recognized treatment for massive rotator cuff tears [6–10]. It relieves pain and improves shoulder function leading to a better quality of life for patients [8]. Although most patients who have undergone RTSA have had good clinical results, some have had poor outcomes and high rates of complications, reoperations, and revisions [11, 12]. These poor outcomes were dependent on several factors including age, gender, previous rotator cuff repair surgery, and preoperative functional scores such as the American Shoulder and Elbow Surgeons (ASES) score [11, 13–16]. Additionally, surgeons are able to predict patient outcomes of RTSA based on the preoperative stage of the disease [17]. Therefore, a preoperative radiographic assessment of patients developing CTA may help surgeons better understand the correlation between disease severity and outcomes after RTSA. However, it remains unclear whether clinical outcomes after RTSA are dependent on the radiographic stage of massive rotator cuff tears.

Therefore, this prospective multicenter observational study was carried out to investigate whether the disease stage of massive rotator cuff tears influences clinical outcomes after RTSA. We hypothesized that severe stages of massive rotator cuff tears lead to worse outcomes and higher revision rates.

## Materials and methods

### Patient population

In this multicenter, observational study, consecutive patients were enrolled prospectively between December 2007 and August 2011 from five specialized shoulder centers (three in Germany, one in France, and one in Switzerland). All patients underwent RTSA with an inverted bearing for massive rotator cuff tears or CTA after failed conservative treatment or joint-preserving surgery.

All patients gave written informed consent to participation in this study and data publication. The Comité intercantonal d’éthique (Jura, Fribourg, Neuchatel; number 01/2008) granted ethics committee approval for this study in September 2008, and all procedures were in accordance with the Declaration of Helsinki.

### Surgical technique and prosthesis design

Each patient was placed under general anesthesia and operated in beach chair position. The deltopectoral approach was used in 60% of shoulders, and a deltoid split approach was used in 40% of shoulders. For RTSA, the Affinis Inverse Reversed Shoulder Prosthesis System (Mathys Ltd Bettlach, Switzerland) with an inverted soft-on-hard bearing was used. On the humeral side, a monoblock stem was placed with cement in 57% of shoulders and without cement in 43% of shoulders; inlays consisted of cobalt-chromium alloy. On the glenoid side, a 2-peg metaglene coated with titanium plasma spray and calcium phosphate was fixed into the native glenoid with one angular stable locking screw (superiorly) and two lag screws (anteriorly and posteriorly). The glenospheres used were made of ultra-high molecular weight polyethylene and were available in three sizes (36, 39, and 42 mm).

### Clinical and radiographic assessment

Patients were examined clinically and radiographically before surgery and at 24 and > 30 months after surgery. Clinical assessment included the Constant score, the ASES score, and range of motion (ROM) for active abduction [18, 19]. Patient pain and satisfaction were assessed using the visual analog scale (VAS). Finally, all complications were recorded, and survival free from any implant component revision rates were calculated.

Radiographic images were taken according to a standard procedure. Each patient stood in a normal upright position, turned approximately 30° toward the involved side with the arm abducted at 30°. True anteroposterior images were taken during expiration to get an orthograde view of the metaglene as described previously [20].

**Fig. 1:**
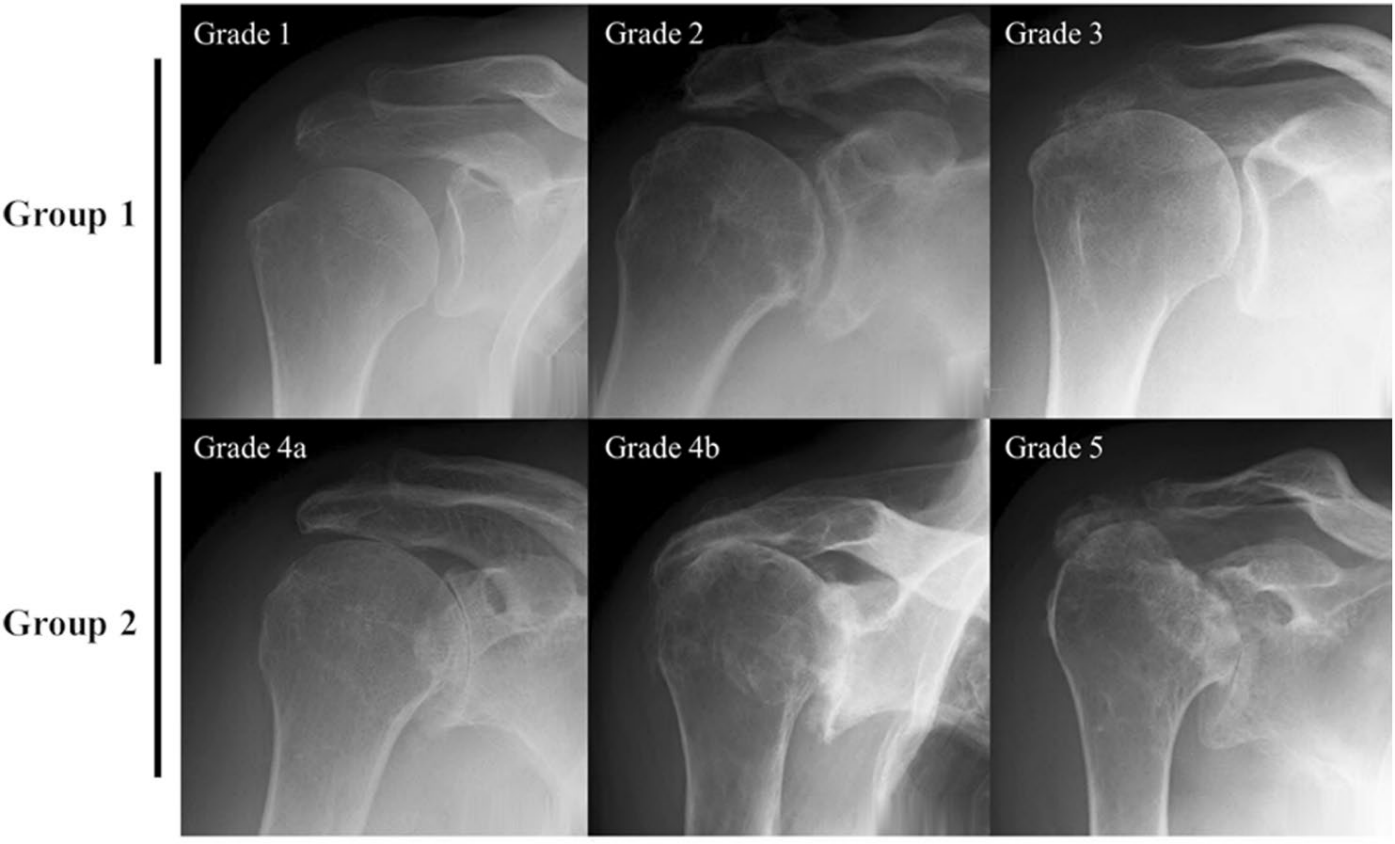
Radiographic images of Hamada classification grades

Each shoulder was assessed by the operating surgeon according to the Hamada classification of the preoperative radiograph and confirmed by an experienced surgeon at each site (Fig. 1). Shoulders without glenohumeral arthritis (Hamada grades 1, 2, and 3) were included in group 1 (low-grade disease). Shoulders with glenohumeral arthritis (Hamada grades 4a, 4b, and 5) were included in group 2 (high-grade disease).

### Statistical analysis

Statistical analysis was performed with the Statistical Analysis System Enterprise Guide, version 7.13 (SAS Institute Inc, Cary, NC, USA). Data were stored on MEMdoc (Institute of Social and Preventative Medicine, University of Berne, Switzerland). Descriptive statistics included means, standard deviations, and ranges. Categorical data were reported as frequencies and percentages. The nonparametric Kruskal–Wallis test and Chi-square test were used to determine differences in baseline characteristics and clinical outcomes between both Hamada groups. The Spearman rank correlation was performed to determine the correlation of Hamada stage with clinical outcomes. Prosthesis survival was analyzed using the Kaplan–Meier method, in which patients were censored at death or when lost to follow-up. Final follow-up was defined as the last date when a patient was clinically and/or radiologically evaluated. The log-rank test was used to compare implant component survival. The level of significance was set at a *P* value of < 0.05 (two-sided).

## Results

### Patient demographics

In total, 202 patients (211 shoulders; 142 in women and 69 in men) with RTSA for massive rotator cuff tears or CTA were treated. Patients had a mean age of 75.8 ± 6.6 years (range 41.9–91.6 years) at the time of surgery. During the study period, 19 patients (20 shoulders) died, four patients (four shoulders) were revised, and 35 patients (36 shoulders) were lost to follow-up. The remaining 144 patients (151 shoulders) were available for the final follow-up examination at a mean of 79.9 ± 24.7 months (range 30.2–132.3 months).

Except for the American Society of Anesthesiologists classification, which was higher in group 2 than in group 1 (*P* = 0.018), baseline characteristics between the 2 groups were similar (*P* ≥ 0.05) (Table 1).

**Table 1:** Baseline characteristics according to Hamada group

Characteristic	Group 1 (*n* = 108)	Group 2 (*n* = 103)	*P* value
Age at surgery mean ± SD (range) years	74.3 ± 6.9 (41.9–91.6)	75.7 ± 6.2 (55.3–87.3)	0.089
Gender, *n* (%)			
Male	40 (37)	29 (28)	0.17
Female	68 (63)	74 (72)	
Hamada classification, *n* (%)			
Grade 1	13	–	
Grade 2	52	–	
Grade 3	43	–	
Grade 4a	–	44	
Grade 4b	–	50	
Grade 5	–	9	
Rheumatoid arthritis, *n* (%)			
Yes	2 (2)	4 (4)	0.37
No	106 (98)	99 (96)	
ASA classification, *n* (%)			
ASA I	12 (11)	6 (6)	0.018*
ASA II	35 (32)	29 (28)	
ASA III +	46 (43)	64 (62)	
Unknown	15 (14)	4 (4)	
Surgical approach, *n* (%)			
Deltopectoral	70 (65)	56 (54)	0.12
Deltoid split	38 (35)	47 (46)	
Follow-up, *n* (%)			
24 months	53 (49)	44 (43)	0.96
> 30 months	83 (77)	68 (66)	0.30

*n* refers to the number of shoulders

*ASA* American Society of Anesthesiologists, *SD* standard deviation

*Statistically significant

Bold value are statistically significant

### Clinical outcomes

The mean Constant score and ASES score improved from preoperative values in each group but remained similar within the groups at both 24 months and the final follow-up examination (*P* > 0.05). Moreover, the clinical scores did not differ significantly between the two groups over the entire observation period (*P* > 0.05) (Table 2). Furthermore, the five Hamada grades did not correlate with clinical outcomes at 24 (*P* = 0.98) and > 30 months (*P* = 0.29). At the final follow-up examination, the mean Constant score reached 63.4 ± 17.4 in group 1 and 62.6 ± 15.6 in group 2 (*P* = 0.33), and the mean ASES score was 79.7 ± 18.1 in group 1 and 75.3 ± 22.0 in group 2 (*P* = 0.17).

**Table 2:** Clinical outcomes according to Hamada group

Outcome	Follow-up		
	Preoperative	24 months	> 30 months
Constant score (points)			
Group 1	26.2 (14.4)	66.8 (15.7)	63.4 (17.4)
Group 2	23.8 (13.2)	68.0 (14.0)	62.6 (15.6)
*P* value	0.25	0.82	0.33
ASES score (points)			
Group 1	20.2	76.4 (17.1)	79.7 (18.1)
Group 2	20.0	79.2 (16.4)	75.3 (22.0)
*P* value	0.82	0.41	0.17
ROM for abduction (°)			
Group 1	71.3 (38.4)	128.7 (40.3)	132.5 (36.4)
Group 2	62.9 (36.2)	133.6 (34.0)	124.6 (36.0)
*P* value	0.12	0.73	0.14
VAS for pain			
Group 1	7.9 (1.7)	1.2 (1.5)	0.9 (1.6)
Group 2	7.7 (1.8)	0.8 (1.4)	1.4 (2.4)
*P* value	0.63	0.18	0.26
VAS for satisfaction			
Group 1	1.9 (1.6)	8.9 (1.3)	9.0 (1.6)
Group 2	2.1 (1.9)	9.2 (1.1)	8.5 (2.5)
*P* value	0.48	0.14	0.55

Values given as means (standard deviations)

*ASES* American Shoulder and Elbow Surgeons, *VAS* visual analog scale, *ROM* range of motion

The ROM for abduction as well as the VAS for patient pain and satisfaction also improved postoperatively compared with preoperative values and remained similar within each group at both 24 months and at the final follow-up examination. Additionally, they did not differ significantly between the groups over the entire observation period (*P* > 0.05).

### Implant survival and complications

One or more postoperative complications were seen in 11 patients (11 shoulders), of which ten underwent revision surgery (four before the final follow-up examination and six thereafter). Five patients were revised to a cemented stem with inlay and glenosphere replacement (three for aseptic stem loosening, one for a periprosthetic fracture, and one for both aseptic stem loosening and periprosthetic fracture). Three patients were converted to a hemiprosthesis, leaving the stem in place (two for a breakout of the metaglene after a fall and one for deep infection with glenoid loosening). Two patients required component revision with glenosphere and inlay exchange (one for persistent deep infection and one for chronic shoulder dislocation). Finally, one patient received closed reduction after shoulder dislocation.

**Fig. 2:**
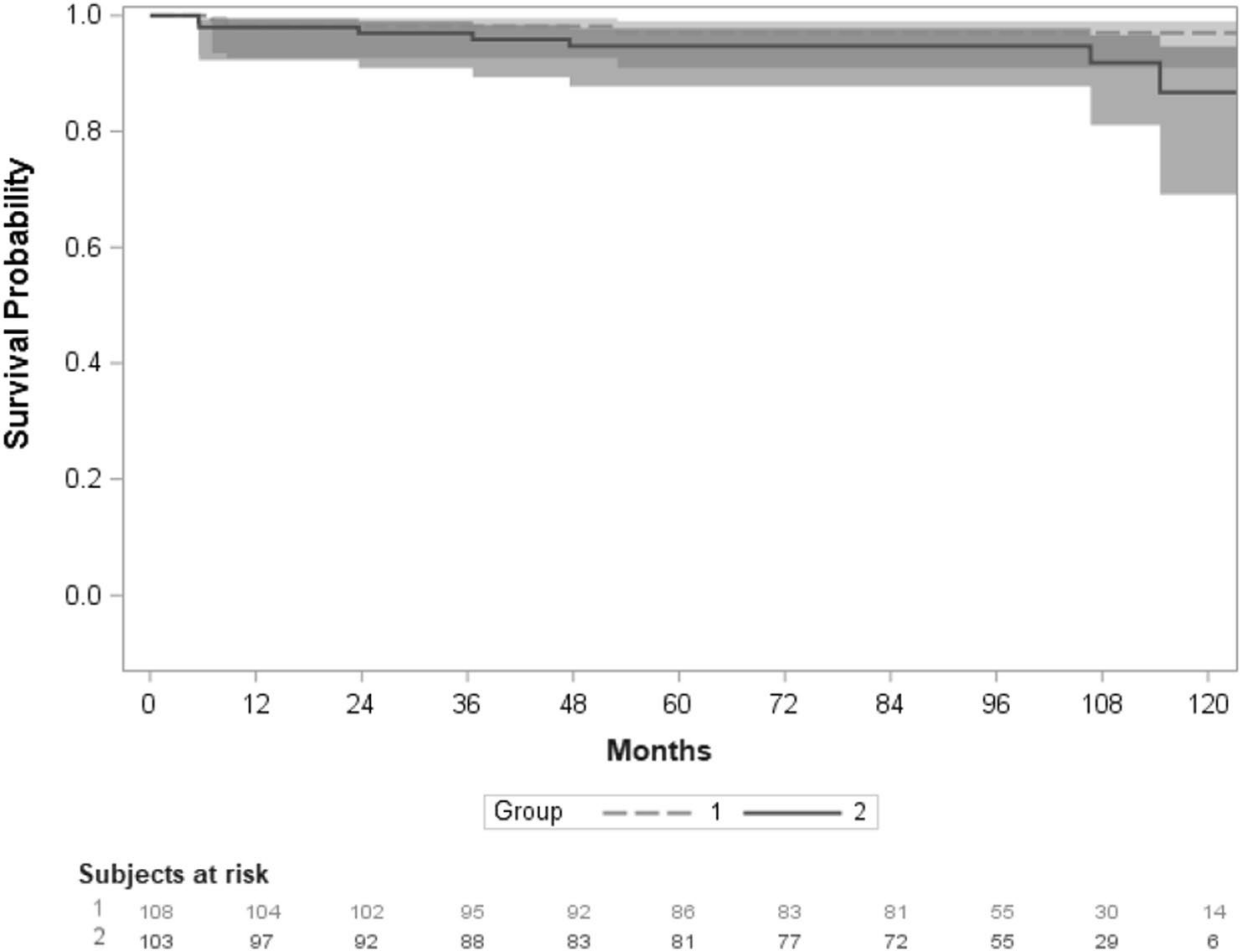
Kaplan–Meier survivorship analysis for survival free from any implant component revision according to Hamada group

Implant survival of any component was similar between both groups (*P* = 0.17) (Fig. 2). Three shoulders (1.4%) in group 1 and seven shoulders (3.3%) in group 2 required component revision.

## Discussion

This study determined whether the stage of rotator cuff tear influenced clinical outcomes after RTSA with an inverted bearing. The midterm results of this study did not confirm our hypothesis that a more severe stage of massive rotator cuff tear would lead to worse outcomes and a higher revision rate. In fact, the Hamada classification did not influence outcomes or revision rates after RTSA.

Although preoperative predictors such as a young age and high preoperative function have been linked to poor functional outcomes [21], there is limited evidence that radiographic stage of massive rotator cuff tears can predict outcomes after RTSA. Similar to our results, recent research found no association between outcomes after RTSA and disease severity as classified by Hamada, Favard, and Walch, nor have researchers linked preoperative factors with an increased incidence of scapular spine fractures after RTSA [22]. Regarding complications, Ernstbrunner et al. found nonsignificant differences in complication rates between shoulders without glenohumeral arthritis (Hamada grades 1, 2, and 3) and those with glenohumeral arthritis (Hamada grades 4 and 5) (56% versus 29%, *P* = 0.38), which aligns with the findings of this study [23]. Taken together, these results reveal no correlation between clinical outcomes and disease severity as ranked by Hamada classification.

Regarding implant type, the results of this study were comparable to those of similar prostheses used for the same indication. First, the functional outcomes of the patients included in this study were consistent with the mean Constant scores reported in the literature [7, 24–26]. Second, the revision rate in this study (4.7%) was lower than that reported for similar prostheses both over comparable (7.3–25%) and longer follow-up periods (45%) [7, 24, 27]. Lastly, the patients in this study had high midterm implant survival rates that were within the range of recently published reports [25, 28].

This study’s main strengths were its midterm follow-up and multicenter setup using a reverse shoulder prosthesis with a soft-on-hard bearing couple. Additionally, the patient cohort was homogenous in terms of indication, treatment, and implant used. Nevertheless, this study faced some limitations. First, a substantial number of patients were lost to follow-up, which can be explained partly by patient age and comorbidities. However, the rate of losses to follow-up was comparable across both groups. Second, multiple orthopedic surgeons were involved in radiographic analysis and classification. To minimize intra- and interobserver variability, all radiographic classifications were confirmed by an experienced surgeon at each site. Moreover, as reported in the published literature, intra- and interobserver variability for the Hamada classification is considered acceptable and in line with or better than other classification systems [4, 5]. Third, the surgical technique was heterogenous; patients were treated with either the deltoid or deltopectoral approach and received cemented and uncemented implants, a situation that reflects typical clinical practice. Lastly, while the Hamada classification reports on glenohumeral joint degeneration, it does not consider morphologic changes in the glenoid and thus may be more useful to classify early stages of massive rotator cuff tears [4, 5]. Later stages of the disease may be better addressed using the Seebauer, Sirveaux, or Favard classifications [2, 3, 29]. Furthermore, some authors have questioned the clinical value of the Hamada classification citing the availability of more modern imaging technologies such as magnetic resonance imaging [4]. Still, the Hamada classification remains the most widely used grading system with acceptable radiation exposure and reliability in everyday practice [5].

## Conclusions

RTSA with an inverted bearing was found to be an effective treatment option with similarly good midterm clinical outcomes, similar revision rates, and high implant survival rates in all disease stages of massive rotator cuff tears. Overall, the preoperative Hamada classification did not influence clinical outcomes or complications after RTSA.
